# Retrochalcone Echinatin Triggers Apoptosis of Esophageal Squamous Cell Carcinoma via ROS- and ER Stress-Mediated Signaling Pathways

**DOI:** 10.3390/molecules24224055

**Published:** 2019-11-09

**Authors:** Ah-Won Kwak, Joon-Seok Choi, Mee-Hyun Lee, Ha-Na Oh, Seung-Sik Cho, Goo Yoon, Kangdong Liu, Jung-Il Chae, Jung-Hyun Shim

**Affiliations:** 1 Department of Pharmacy, College of Pharmacy, Mokpo National University, Jeonnam 58554, Korea; rhkrdkdnjs12@mokpo.ac.kr (A.-W.K.); 17392303@mokpo.ac.kr (H.-N.O.); sscho@mokpo.ac.kr (S.-S.C.); gyoon@mokpo.ac.kr (G.Y.); 2 College of Pharmacy, Daegu Catholic University, Havang-Ro 13-13, Havang-Eup, Gyeongsan-si, Gyeongbuk 38430, Korea; joonschoi@cu.ac.kr; 3 The China-US (Henan) Hormel Cancer Institute, Zhengzhou 450008, Henan, Chinakangdongliu@126.com (K.L.); 4 Basic Medical College, Zhengzhou University, Zhengzhou 450001, Henan, China; 5 Department of Dental Pharmacology, School of Dentistry, BK21 Plus, Jeonbuk National University, Jeonju 54896, Korea

**Keywords:** Esophageal squamous cell carcinoma, Echinatin, Reactive oxygen species, c-Jun N-terminal kinase, p38

## Abstract

Esophageal squamous cell carcinoma (ESCC) is a poor prognostic cancer with a low five-year survival rate. Echinatin (Ech) is a retrochalone from licorice. It has been used as a herbal medicine due to its anti-inflammatory and anti-oxidative effects. However, its anticancer activity or underlying mechanism has not been elucidated yet. Thus, the objective of this study was to investigate the anti-tumor activity of Ech on ESCC by inducing ROS and ER stress dependent apoptosis. Ech inhibited ESCC cell growth in anchorage-dependent and independent analysis. Treatment with Ech induced G2/M phase of cell cycle and apoptosis of ESCC cells. It also regulated their related protein markers including p21, p27, cyclin B1, and cdc2. Ech also led to phosphorylation of JNK and p38. Regarding ROS and ER stress formation associated with apoptosis, we found that Ech increased ROS production, whereas its increase was diminished by NAC treatment. In addition, ER stress proteins were induced by treatment with Ech. Moreover, Ech enhanced MMP dysfunction and caspases activity. Furthermore, it regulated related biomarkers. Taken together, our results suggest that Ech can induce apoptosis in human ESCC cells via ROS/ER stress generation and p38 MAPK/JNK activation.

## 1. Introduction

Esophageal cancer is the one of most common cancer types. In 2018, approximately 321,670 patients in the USA suffer from this cancer, and the total estimated number of deaths from the disease was 13,020 (4%). Its global mortality rate is currently ranked fifth for men and ninth for women [1,2]. There are different types of esophageal cancer, including esophageal squamous cell carcinoma (ESCC: ~90%) and esophageal adenocarcinoma (EA: ~10%) [3]. In particular, ESCC shows a high incidence rate in Eastern Europe, South America, Eastern/Central Asia, and Eastern/Southern Africa. Its five-year survival rate is only ~10% for high grade occurrences [3]. Chemotherapy against ESCC is available with cisplatin, paclitaxel, or 5-fluorouracil. However, these therapies have side effects, including toxicities that are difficult issues to solve. Since natural compounds show benefits such as reduced toxicity, they might be useful for ESCC treatment [4]. Natural compounds such as herbal medicines can inhibit various cancers including lung, colon, oral, prostate, and ovarian cancers [5,6,7,8]. Echinatin (Ech) is a retrochalcone compound. It is a main component of licorice root extracts, particularly from Glycyrrhiza inflata species [9]. The pharmacological activities of Ech occur due to its antioxidative and anti-inflammatory effects [9,10]. Ech can induce about a four-fold increase of activity of the nuclear factor erythroid 2-related factor 2, a transcription factor that transcribes antioxidant proteins, thereby protecting mice against hepatoxicity and carbon tetrachloride-induced acute liver injury [10]. In another study, lipopolysaccharide treated RAW 264.7 macrophages showed higher levels of inflammation factors with the induction of prostaglandin E2 and interleukin-6, whereas Ech reduced these inflammatory factors’ production [9]. Cancer cell death induced by anticancer drug is caused by intrinsic or extrinsic apoptosis signaling pathways including Bcl-2/Bax/Bid/cytochrome C (cyto C)/apoptotic protease activating factor-1 (Apaf-1)/caspase-9 or death receptors (DR)/caspase-8/Bid together with executional activity of caspase-3 and caspase-7 [11,12]. In addition, reactive oxygen species (ROS), endoplasmic reticulum (ER) stresses, and stress-inducible proteins such as c-Jun N-terminal kinases (JNK) and p38 can induce cell apoptosis through intrinsic cell death signaling pathways [13,14,15].

However, the anticancer activity of Ech has been rarely studied. Therefore, the objective of this study was to determine whether Ech might have inhibitory effects on ESCC cell growth. In this study, we found that Ech inhibited cell growth and induced cell cycle at the G2/M phase and apoptosis in ESCC cell lines. We then examined the mechanism involved in the apoptosis induced by Ech in detail by detecting ROS level, ER stress, and their biomarkers. Our results revealed that Ech triggered ROS production and 78 kDa glucose-regulated protein (GRP78), CCAAT/enhancer-binding protein homologous protein (CHOP), DR4, and DR5 expression. It also activated phospho-JNK (p-JNK) and phospho-p38 (p-p38), thus inducing apoptosis of ESCC cells by both intrinsic and extrinsic cell death signaling pathways.

## 2. Results

### 2.1. Ech Inhibits ESCC Cell Growth

**Figure 1:**
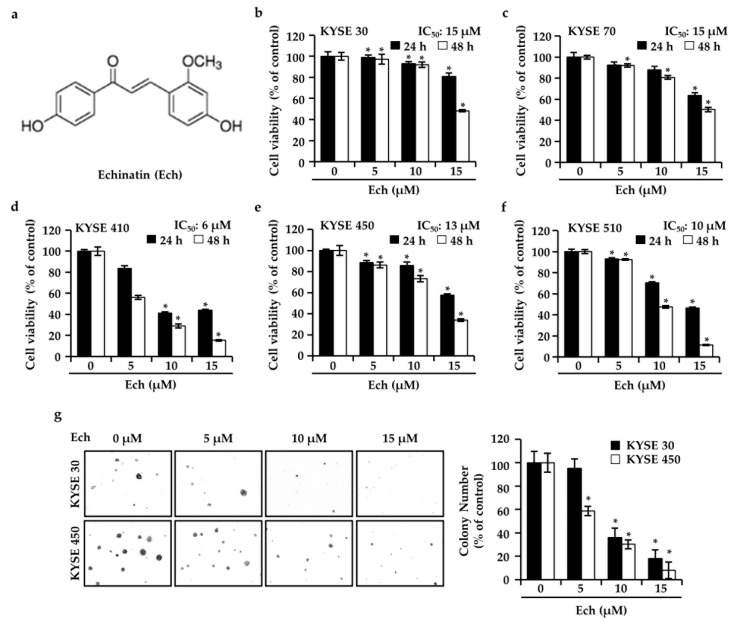
Effects of echinatin (Ech) on cell growth. (**a**) Structure of Ech. (**b–f**) Ech inhibited KYSE 30, KYSE 70, KYSE 410, KYSE 450, and KYSE 510 ESCC cells growth at 5, 10, or 15 µM for 24 h or 48 h. Cell viability was examined using MTT assay. (**g**) Representative pictures (left panel) and graph (right panel) of KYSE 30 and KYSE 450 cells colonies treated with Ech (0, 5, 10, 15 µM) for 14 days. Colonies were counted using a microscope. The graph shows the percentage compared to the control group. Data represent mean ± SD. Asterisk (*) denotes *p* < 0.05 compared to the control.

At the beginning, we examined effects of Ech (Figure 1a) on ESCC cells (Figure 1). Ech significantly inhibited growth of ESCC cells in a dose- and time- dependent manner compared to control (Figure 1b–f). The concentration for inhibiting 50% of cell growth (IC_50_) of Ech was 15 µM for KYSE 30, 15 µM for KYSE 70, 6 µM for KYSE 410, 13 µM for KYSE 450, and 10 µM for KYSE 510. In the three-dimensional culture of ESCC cells, colony numbers and sizes were decreased by treatment with Ech in KYSE 30 and KYSE 450 cells (Figure 1g). Ech at 5, 10, and 15 µM inhibited KYSE 30 cell colonies by 4.92%, 44.26%, and 81.97%, respectively. Similarly, Ech at 5, 10, and 15 µM inhibited KYSE 450 cell colonies by 41.27%, 69.84%, and 92.06%, respectively.

### 2.2. Ech Arrests Cell Cycle of ESCC Cells at G2/M Phase and Induces Apoptosis

**Figure 2:**
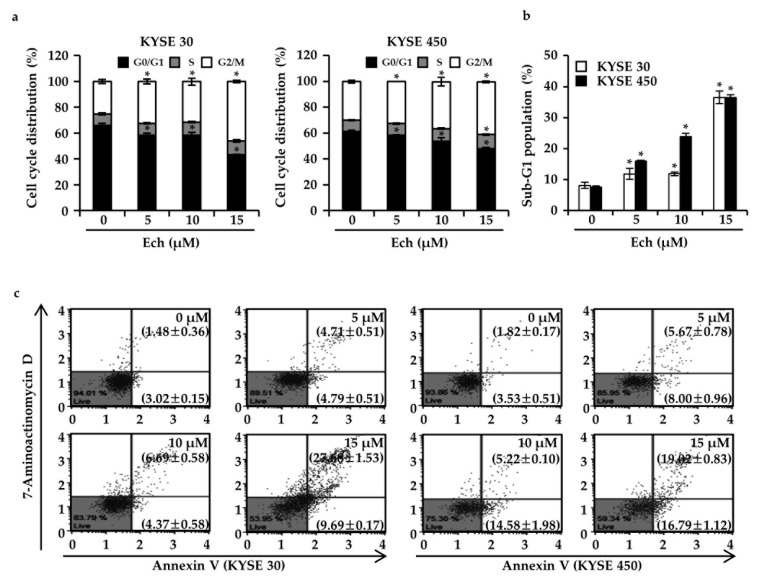
Effects of Ech on cell cycles and apoptosis. (**a**) Ech arrested G2/M phase of cell cycle and (**b**) induced sub-G1 population in KYSE 30 and KYSE 450 cells. (**c**) Ech increased apoptotic population of KYSE 30 and KYSE 450 cells. Viable cells (Annexin V negative/7-AAD negative) are shown in the lower left; Early apoptotic cells (Annexin V positive/7-AAD negative) are shown in the lower right; Late apoptotic cells (Annexin V positive/7-AAD positive) are shown in the upper right; Necrotic cells (Annexin V negative/7-AAD positive) are shown in the upper left. Cells were treated with Ech at 0, 5, 10, or 15 µM for 48 h, stained with 7-AAD for the cell cycle or Annexin V/7-AAD for apoptosis, and analyzed with Muse™ Cell Analyzer. Asterisk (*) denotes *p* < 0.05 compared to the control.

**Figure 3:**
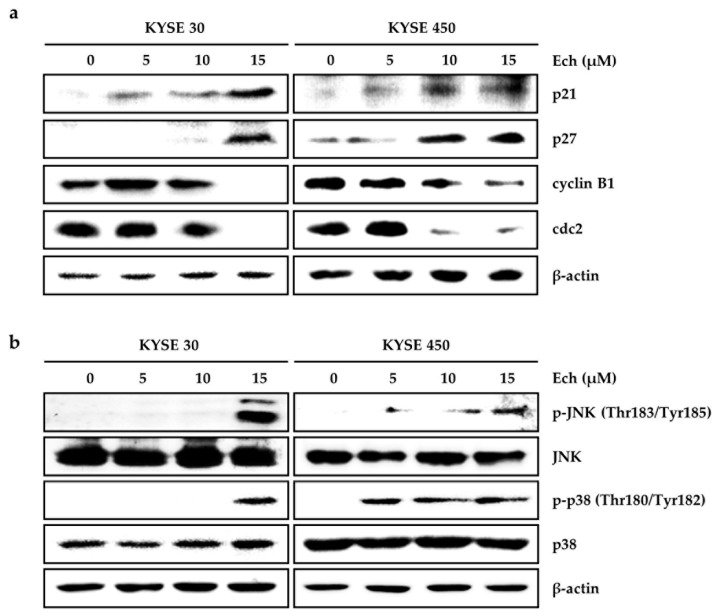
Effects of Ech on cell cycle and cell death related signals. (**a**) Ech induced p21 and p27 expression but decreased cyclin B1 and cdc2 expression. (**b**) Ech induced p-JNK and p-p38 expression, although total proteins levels of JNK or p38 were not changed. KYSE 30 and KYSE 450 cells were treated with Ech (0, 5, 10, 15 µM) for 48 h. The expression was examined with Western blot. β-actin was used as a loading control.

Cell growth processes contain the cell cycle’s promotion [16]. Thus, Ech may affect the cell cycle and cause ESCC cell growth inhibition. When we treated KYSE 30 and KYSE 450 ESCC cells with Ech at 0, 5, 10, or 15 µM, cell cycles were accumulated at G2/M phase compared to control (Figure 2a). Sub-G1 population was dose-dependently increased by Ech (increase after treatment with Ech at 0, 5, 10, or 15 µM: 8.17 ± 0.99, 11.83 ± 1.78, 11.87 ± 0.55, and 36.53 ± 2.02% in KYSE 30 cells; 7.57 ± 0.47, 15.97 ± 0.25, 23.80 ± 1.15, and 36.47 ± 0.93% in KYSE 450 cells, respectively) (Figure 2b). Sub-G1 death cells can be caused by apoptosis or necrosis [17]. Thus, we stained cells with Annexin V for apoptosis or 7-Aminoactinomycin D (7-AAD) for necrosis (Figure 2c). Early apoptosis percentage of Annexin V+/7-AAD- gating was increased to 9.69 ± 0.17% or 16.79 ± 1.12%, while the late apoptosis percentage of Annexin V+/7-AAD+ gating was increased to 27.68 ± 1.53 or 19.02 ± 0.83% in KYSE 30 or KYSE 450 ESCC cells after treatment with 15 µM Ech, respectively (Figure 2c). To verify the effects of Ech on cell cycle and apoptosis, we conducted Western blot to examine expression of the cell cycle at G2/M phase and apoptosis signaling markers (Figure 3a,b). After KYSE 30 and KYSE 450, cells were treated with Ech at 5, 10, or 15 µM for 48 h, expression levels of cell cycle markers p21 and p27 were increased while those of cyclin B1 and cdc2 were decreased compared the control (Figure 3a). For apoptosis signaling markers, Ech induced expression levels of p-JNK and p-p38 mitogen-activated protein kinase (MAPK) (compared to total form of JNK and p38, respectively) using β-actin as control (Figure 3b).

### 2.3. Ech Induces Apoptosis by Increasing ROS Levels and ER Stress

**Figure 4:**
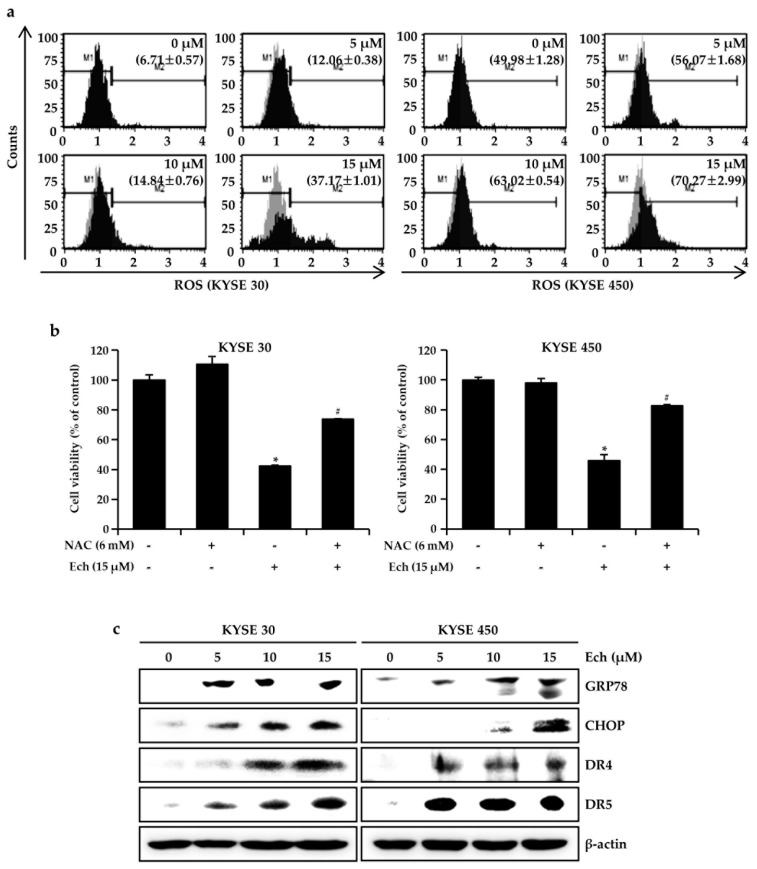
Effects of Ech on intracellular ROS induction. (**a**) Ech induced ROS formation in ESCC cells. ROS formation was measured with a Muse™ Cell Analyzer (materials and method). M1 refers to the fraction of ROS negative population while M2 refers to the region of ROS positive population. Average percentages of ROS positive cells are shown in the M2 region. (**b**) NAC rescued Ech induced cell death through ROS scavenging. Cells were pretreated with 6 mM NAC for 3 h and then exposed to 15 μM Ech for 48 h. Data are presented as mean ± SD of three independent experiments (* *p* < 0.05 vs. untreated control; # *p* < 0.05 vs. Ech-treated cells). (**c**) Ech increased ROS-mediated signal proteins. Expression levels of GRP78, CHOP, DR4, and DR5 were determined with Western blot. β-actin was used as a loading control.

To determine the increase of p-p38 and p-JNK expression via induction of ROS, we detected ROS levels after treatment with dimethyl sulfoxide (DMSO) as a control and Ech (5, 10, 15 µM) for 48 h (Figure 4a). Ech at 0, 5, 10, and 15 µM induced ROS levels by 6.71 ± 0.57, 12.06 ± 0.38, 14.84 ± 0.76, and 37.17 ± 1.01% in KYSE 30 cells, as well as 49.98 ± 1.28, 56.07 ± 1.68, 63.02 ± 0.54, and 70.27 ± 2.99% in KYSE 450 cells, respectively. To confirm the involvement of ROS in apoptosis induction, we measured viabilities of KYSE 30 and KYSE 450 cells treated with a combination of ROS scavenger N-acetyl-l-cysteine (NAC, 6 mM) and Ech (15 µM) (Figure 4b). Results revealed that treatment with NAC only did not significantly affect the viabilities of either cell line. However, Ech significantly decreased the viabilities of KYSE 30 and KYSE 450 cells. Its decreases were recovered by NAC treatment (Figure 4b). To further determine whether Ech-induced ROS could activate ER stress cascades, thereby inducing apoptosis of ESCC cells, we examined expression levels of ER stress related proteins (Figure 4c). Ech induced DR4 and DR5 expression in KYSE 30 and KYSE 450 cells in a dose-dependent manner (Figure 4c). Expression levels of GRP78 and CHOP, down-stream targets of DR4 and DR5 proteins, were also increased by Ech in a dose-dependent manner compared to those in the control (DMSO treated).

### 2.4. Ech Provokes Mitochondrial Dysfunction and Caspases Activation

**Figure 5:**
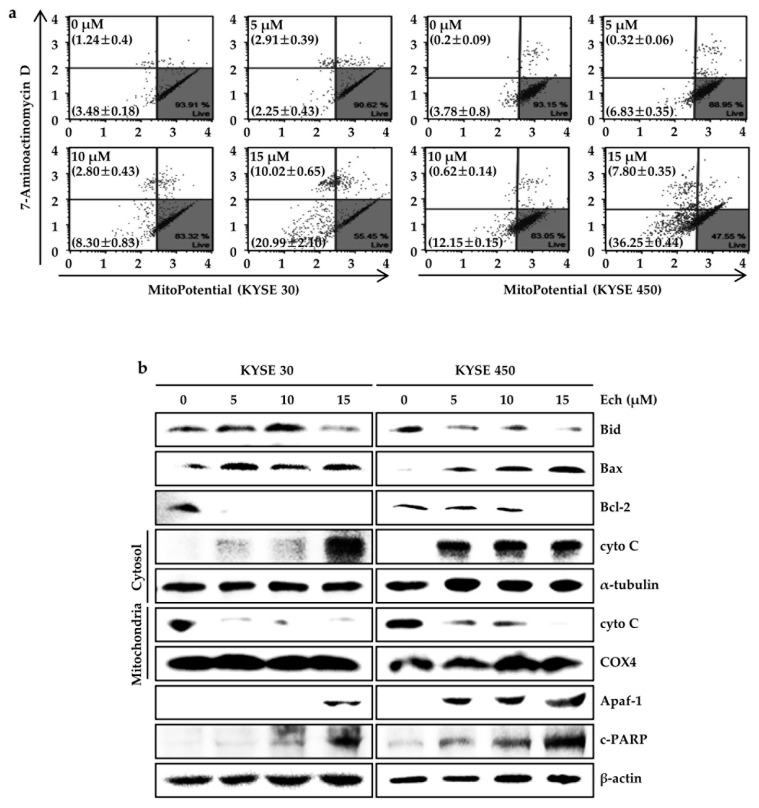
Effect of Ech on MMP activities and apoptosis-related proteins. (**a**) Ech induced MMP dysfunction. Cells were treated with Ech (0, 5, 10, or 15 µM) for 48 h, stained with MitoPotential Dye and 7-AAD, and analyzed with a Muse™ Cell Analyzer. Percentages in the left top show depolarized/dead cells. Percentages in the left bottom show depolarized/live cells. Experiments were done at least three times in triplicate. Data are presented as means ± SD. (**b**) Ech decreased Bid, Bcl-2, and cyto C (mitochondria) expression but increased Bax, cyto C (cytosol), Apaf-1, and c-PARP expression. Western blotting was performed to determine expression levels. β-actin, α-tubulin (cytosol), and COX4 (mitochondria) were used as loading controls.

**Figure 6:**
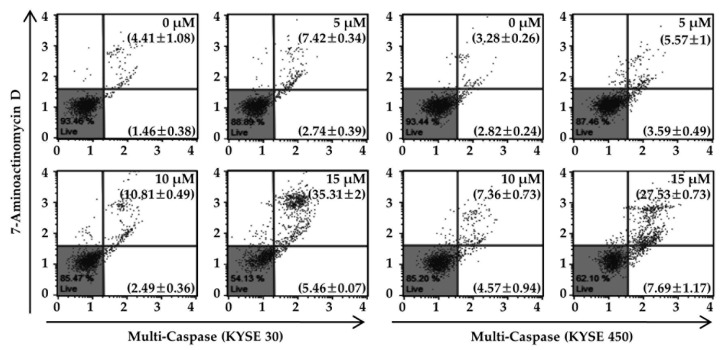
Effects of Ech on multi-caspases activity in KYSE 30 and KYSE 450 cells. After cells were treated with Ech at various concentrations for 48 h, percentages of KYSE 30 and KYSE 450 cells with caspases activities were assessed. *X*-axis means the degree of caspase activity and *y*-axis shows the fluorescence of 7-AAD. Each proportion in the figure represents multi-caspases positive/live cells (lower right) or multi-caspases positive/dead cells (upper right). All tests were performed in triplicate. Values are expressed as mean ± SD.

Next, we determined whether the induction of ROS level and ER stress caused by Ech treatment influenced mitochondrial membrane potential (MMP) dysfunction (Figure 5). Ech at 15 µM obviously induced depolarization of MMP in KYSE 30 and KYSE 450 ESCC cells by 31.01 ± 1.72 and 44.05 ± 0.43%, respectively (Figure 5a). Mitochondria-mediated apoptosis markers were also regulated by Ech treatment (Figure 5b). Ech decreased expression of Bid, and Bcl-2 but increased Bax expression. In addition, Ech resulted in the release of cyto C from mitochondria to the cytosol in a dose-dependent manner compared to the DMSO control and α-tubulin and COX-4 control fraction proteins (Figure 5b). Expression levels of the following signal proteins of apoptosis, Apaf-1 and cleaved Poly (ADP-Ribose) Polymerase (c-PARP), were increased by treatment with Ech in a dose-dependent manner compared to those in the control. We also measured activities of caspases, an upstream protein of PARP, and a downstream protein of Apaf-1 in apoptosis signaling pathway (Figure 6).

## 3. Discussion

**Figure 7:**
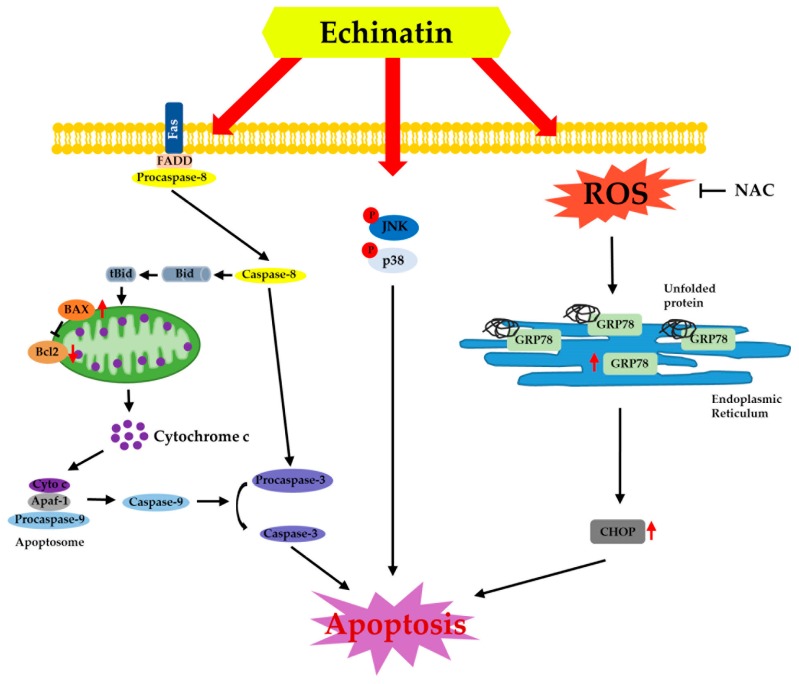
Schematic showing Ech-induced cell death. Ech induces ROS production and activates p38 and JNK MAPK kinases, or stimulates ER stress. p38 and JNK MAPK kinases signaling cascades increase death receptor expression and caspase-8/Bid activation, BAX expression, Bcl2 reduction, and cytochrome c release (cytosol), which then induces Apaf-1/caspase9 or caspase-3 activation, thus resulting in cell apoptosis. On the other hand, ER stress with increasing GRP78 and CHOP expression will induce cancer cell apoptosis.

Chemotherapy of ESCC still needs further investigation to obtain proper efficiency with low side effects [18]. Natural medicines such as aspirin from willow bark and eupatilin from artemisia have low side effects. They have been used for treating cancers [19]. Here, we examined the anticancer effect of Ech from *Glycyrrhiza inflata* (licorice) on ESCC and found that it had inhibitory activities against ESSC cells. Although other components of licorice such as licochalcones A, B, and C have shown anticancer effects on colon, skin, and oral cancers [20,21,22], the anticancer effect of Ech has not been well elucidated yet. Many anticancer agents can inhibit cancer cell proliferation by arresting cell cycle at G1- or G2/M-phase [23]. The G2 checkpoint can prevent cells from entering mitosis when DNA is damaged. It ensures the propagation of error-free copies of the genome to each daughter cell. Cdk1/cyclin B1 complex controls the cell cycle progression from the G2 phase to the M phase by regulating phosphorylation or dephosphorylation of proteins [24]. In addition, actin remodeling in coordination can ensure proper execution of G2/M checkpoint arrest. It is crucial for entry into mitosis. Flow-cytometry analysis results of the present study revealed that Ech induced G2/M phase arrest of cell cycle (Figure 2a). We then detected expression of related markers, including cyclin B1, cdc2 (Cdk1), and p27 (Figure 3a). Increase of p27 expression regulates cell cycle at G2/M and suppresses cdc2/cyclin B1 expression [25]. Apoptosis is a type I cell death. It physiologically shows plasma membrane shrinkage and nuclear fragmentation following death ligands (extrinsic pathway) and DNA damage/cell stresses (intrinsic pathway) [26]. Cell stresses caused by chemotherapeutic agents can stimulate stress activated MAPKs including p38 MAPK and JNK [15]. Ech induced activation of p38 MAPK and JNK based on the detection of their phosphorylation forms by Western blotting (Figure 3b). Accumulating evidence suggests that protein folding and generation of ROS as a byproduct of protein oxidation in the ER are closely linked to each other [27]. ROS have emerged as crucial regulators of ER function in several diseases. Induction of ER stress and ROS production occur concurrently. GRP78 and CHOP are commonly used as markers of ER stress. GRP78, the master regulator of the unfolded protein response (UPR), plays a role in proliferation, invasion, and metastasis in cancer [28]. GRP78 represents both a regulator and a target of the UPR. It is associated with pro-survival responses. Conversely, GRP78 can interact with components of ER related pro-apoptotic pathways [29]. In a previous study, only GRP78 negative colon cancer cells were found to be highly proliferative. They induced significant growth in tumor size and metastasized to the liver [30]. In contrast, GRP78 positive cells manifested reduced proliferation, colony formation, tumor growth, and liver metastases [30]. CHOP as a transcription factor is also involved in ER stress-induced apoptosis [31]. Ech induced ROS formation, ER stress, and expression of DR4, DR5, GRP78, and CHOP biomarkers (Figure 4). We also confirmed Ech-induced cell death via ROS through treatment with NAC (Figure 4b). ER stress and ROS formation can induce mitochondrial potential disruption and result in apoptosis [32,33]. Treatment of ESCC cells with Ech induced MMP dysfunction and released cyto C from mitochondria to cytosol, thereby activating caspases (Figure 5 and Figure 6). In summary, Ech inhibited ESCC cell growth by inducing intrinsic and extrinsic apoptosis pathways through ROS- and ER-stress mediated signaling (Figure 7). At present, phase I or phase II clinical trials using natural compounds such as resveratrol and grape powder (NCT 00256334, NCT 01370889), ginger root extract (NCT 01344538), and sulforaphane (NCT 01228084) for colorectal cancer or prostate cancer have been conducted or are being conducted. A successful natural medicine with clear evaluation and prediction in clinic is needed. Thus, it is necessary to investigate the action mechanisms of these compounds in detail.

## 4. Materials and Methods 

### 4.1. Purification of Echinatin

Air-dried, powdered G. inflata roots (2 Kg) were purchased from Chonnam Herb Association and identified by Prof. Gil-Saeng Jeong of the College of Pharmacy, Keimyung University. They were then extracted twice with MeOH (4 L) by sonication for 3 h. After filtration, the solvent was evaporated in vacuo to provide a MeOH extract (170 g) which was suspended in H2O (1 L) and extracted successively with n-hexane, methylene chloride, and EtOAc. The methylene chloride extract (9 g) was subjected to flash silica gel chromatography using a n-hexane:EtOAc solvent system (2:1~1:1) to give 10 fractions. Fraction 6 was subjected to further flash silica gel chromatography with a chloroform:MeOH (10:1) eluent system to afford echinatin (150 mg). Echinatin was further purified by recrystallization with MeOH.

Echinatin (Ech, purity > 95%) characterization data were: ESI-MS *m*/*z*: 270 [M]^+^. (Calcd for C16H14O4 270.08); 1H-NMR (400 MHz, Acetone-d6) δ: 8.11 (2H, d, *J* = 8.4 Hz, H-2′, 6′), 8.07 (1H, d, *J* = 15.6 Hz, H-β), 7.74 (1H, d, *J* = 8.4 Hz, H-6), 7.70 (1H, d, *J* = 15.6 Hz, H-α), 7.00 (2H, d, *J* = 8.4 Hz, H-3′, 5′), 6.58 (1H, d, *J* = 2.0 Hz, H-3), 6.55 (1H, dd, *J* = 2.0, 8.4 Hz, H-5), 3.90 (3H, s, 2-OCH_3_); 13C NMR (400 MHz, Acetone-d6) δ: 186.8 (C = O), 160.6 (C-4′), 160.4 (C-4), 159.5 (C-2), 137.6 (C-β), 129.8 (C-2′), 129.8 (C-6′), 129.7 (C-6), 129.2 (C-1′), 117.5 (C-α), 114.7 (C-1), 114.2 (C-3′), 114.2 (C-5′), 107.1 (C-5), 98.0 (C-3), 54.0 (2-OCH_3_).

### 4.2. Chemicals and Reagents

DMSO, 3-(4,5-dimethylthiazol-2-yl)-2,5-diphenyltetrazolium bromide (MTT), and NAC were bought from Sigma-Aldrich (St. Louis, MO, USA). Cell culture media (RPMI-1640 and DMEM) and supplementary reagents were purchased from Hyclone (Logan, UT, USA) or Welgene (Daegu, Korea). Antibodies against p21, p27, cdc2, cyclin B1, GRP78, CHOP, DR4, DR5, Bid, Bax, Bcl-2, cyto C, α-tubulin, COX-4, Apaf-1, PARP, c-PARP, β-actin, p-JNK (Thr183/Try185), JNK, p-p38 (Thr180/Try182) and p38 were purchased from Santa Cruz Biotechnology (Santa Cruz, CA, USA) or Cell Signaling Technology (Danvers, MA, USA).

### 4.3. Cell Culture

Human ESCC cell lines (KYSE 30, KTYSE 70, KTSE 410, KYSE 450 and KYSE 510) were purchased from the Type Culture Collection of the Chinese Academy of Sciences (Shanghai, China) and the American Type Culture Collection (ATCC, Rockville, MD, USA). Human ESCC cells were maintained in RPMI1640 contained with 10% fetal bovine serum and 1% penicillin/streptomycin. All cells were incubated at 37 °C in a 5% CO_2_ incubator and passaged within 8 times (2 months). 

### 4.4. Cell Growth Assay

ESCC cells were seeded with optimal cell numbers (KYSE 30: 2.75 × 10^3^/well; KYSE 70: 10 × 10^3^/well; KYSE 410: 2.5 × 10^3^/well; KYSE 450: 3.5 × 10^3^/well; and KYSE 510: 5.5 × 10^3^/well) into 96-well plates and incubated at 37 °C overnight. Cells were then treated with 0, 5, 10, or 15 μM of Ech for 24 h or 48 h. Growing cells were detected by adding MTT solution followed by incubation at 37 °C for 1 h. Formazan crystals were then dissolved with DMSO. Absorbance was measured at wavelength of 570 nm with a spectrophotometer (Thermo Fisher Scientific, Vantaa, Finland).

### 4.5. Soft Agar Assay

Bottom layer contained 0.6% agar and basement membrane extract mix. DMSO or Ech (5, 10, 15 μM) was fixed in 6-well plates for 1 h. The top layer included 8 × 10^3^ ESCC cells/well and a mixture of 0.3% agar and basement membrane extract mix with adding DMSO or Ech. Cells were incubated at 37 °C in a 5% CO_2_ incubator for two weeks. Colony numbers were counted using a microscope (Leica Microsystems, Wetzlar, Germany) 

### 4.6. Cell Cycle Analysis

KYSE 30 (7.5 × 10^4^ cells/well) and KYSE 450 (10.5 × 10^4^ cells/well) cells were seeded into 6-well plates and exposed to DMSO or Ech (5, 10, 15 µM) for 48 h. Cells were harvested and washed with 1× phosphate-buffered saline (PBS) three times and then fixed in 70% ethanol at −20 °C overnight. Cells were centrifuged at 4000 rpm for 10 min, washed with 1× PBS three times, and then suspended with 200 μL of Muse™ Cell Cycle Reagent (EMD Millipore, Billerica, MA, USA) for 30 min in the dark. The cell cycle status of cells was analyzed with a Muse™ Cell Analyzer (EMD Millipore, Billerica, MA, USA).

### 4.7. Apoptosis Analysis

Cells (KYSE 30: 7.5 × 10^4^ cells/well; KYSE 450: 10.5 × 10^4^ cells/well) were seeded into 6-well plates and incubated at 37 °C in a 5% CO_2_ incubator overnight. After treatment with DMSO or Ech (5, 10 and 15 µM) for 48 h, ESCC cells were stained with Annexin V and dead cell reagents (Muse™ Apoptosis Assay kit, EMD Millipore). Apoptotic cells were then analyzed using the Muse™ Cell Analyzer.

### 4.8. Western Blotting

Cells were lysed in RIPA buffer (iNtRON, Gyeonggi-do, Korea) for 30 min on ice. Proteins were separated by SDS-PAGE and transferred onto polyvinylidene fluoride membranes (EMD Millipore, Billerica, MA, USA). These membranes were blocked with 3% or 5% skim milk at RT for 2 h and incubated with primary antibodies (dilution, 1:1000) at 4 °C overnight. After removing non-specific binding antibodies with 1× washing buffer (PBS with 0.1% Tween-20) three times, blots were probed with HRP-conjugated secondary antibody (dilution of 1:7000) in skim milk at RT for 2 h. Specific bands were detected using an ImageQuant LAS 500 (GE Healthcare, Uppsala, Sweden).

### 4.9. Cytosolic and Mitochondrial Fractions

ESCC cells (KYSE 30: 2.8 × 10^5^ cells; KYSE 450: 4.4 × 10^5^ cells) were cultured in 100 mm dishes and treated with DMSO or Ech (5, 10, 15 μM) for 48 h. Cells were harvested and mixed with plasma membrane extraction buffer (250 mM sucrose, 10 mM HEPES (pH 8.0), 10 mM KCl, 1.5 mM MgCl_2_•6H_2_O, 1 mM EDTA, 1 mM EGTA, 0.1 mM phenylmethylsulfonyl fluoride, 0.01 mg/mL aprotinin, 0.01 mg/mL leupeptin) and 0.05% digitonin at RT for 1 min. Cytosolic fraction (supernatant) was harvested after centrifugation at 13,000 rpm for 5 min at 4 °C. For the mitochondrial fraction, cell pellets were further suspended with plasma membrane extraction buffer and 0.5% Triton X-100. Suspended pellets were tapped and incubated on ice for 10 min. The mitochondrial fraction was harvested after centrifugation at 13,000 rpm for 30 min. Cyto C expression in the cytosolic or membrane fraction was detected by Western blotting.

### 4.10. Reactive Oxygen Species (ROS) Measurements

Cells (KYSE 30: 7.5 × 10^4^ cells/well; KYSE 450: 10.5 × 10^4^ cells/well) were seeded into 6-well plates. Both KYSE 30 and KYSE 450 cell lines were treated with DMSO or Ech (5, 10, 15 μM) for 48 h. Cells were harvested, suspended in Muse™ Oxidation Stress Reagent working solution (EMD Millipore), and then incubated at 37 °C for 30 min in the dark. ROS levels were measured with the Muse™ Cell Analyzer.

### 4.11. Mitochondrial Membrane Potential (MMP) Assay

KYSE 30 and KYSE 450 cells were treated with DMSO or Ech (5, 10, 15 μM) for 48 h and harvested after centrifuging at 3000 rpm for 5 min at 4 °C. After washing with 1× assay buffer, cells were stained with Mitopotential working solution (Muse™ Mitopotential kit, EMD Millipore) at 37 °C for 20 min in the dark, added with 5 μL of 7-AAD, and incubated at RT for 5 min. MMPs were then observed with the Muse™ Cell Analyzer.

### 4.12. Multi-Caspase Activity Analysis

ESCC cells were treated with DMSO or Ech (5, 10, 15 μM) and harvested to measure multi-caspase activity. Samples were mixed with 1× caspase buffer and 50 μL of Muse™ Multi-Caspase reagent working solution (Muse™ Multi-Caspase kit, EMD Millipore), incubated at 37 °C for 30 min, and then added with 125 μL of Muse™ caspase and 7-AAD for each sample. Multi-caspase stained cells were then analyzed with the Muse™ Cell Analyzer.

### 4.13. Statistical Analysis

Data are presented as means ± standard deviation (SD). All statistical analyses of data were performed using Prism 5.0 statistical package. Statistical significance of differences among groups was analyzed using analysis of variance (ANOVA). Mean values were considered to be significantly different at *p* < 0.05.
